# Spectral Analysis of Chinese Medicinal Herbs Based on Delayed Luminescence

**DOI:** 10.1155/2016/8469024

**Published:** 2016-07-11

**Authors:** Jingxiang Pang, Xiaoyan Zhu, Yanli Liu, Jialei Fu, Xiaolei Zhao, Meina Yang, Eduard van Wijk, Mei Wang, Xiaoyan Nie, Jinxiang Han

**Affiliations:** ^1^Shandong Medicinal Biotechnology Center, Key Laboratory for Biotech-Drugs, Ministry of Health, Shandong Academy of Medical Sciences, Jinan, Shandong 250062, China; ^2^Shandong Center for Disease Control and Prevention, Jinan, Shandong 250014, China; ^3^Department of Basic Medicine, Shandong University of Traditional Chinese Medicine, Jinan, Shandong 250355, China; ^4^Shandong Academy of Traditional Chinese Medicine, Jinan, Shandong 250014, China; ^5^Sino-Dutch Centre for Preventive and Personalized Medicine/Centre for Photonics of Living Systems, Leiden University, 2300 RA Leiden, Netherlands

## Abstract

Traditional Chinese medicine (TCM) plays a critical role in healthcare; however, it lacks scientific evidence to support the multidimensional therapeutic effects. These effects are based on experience, and, to date, there is no advanced tool to evaluate these experience based effects. In the current study, Chinese herbal materials classified with different cold and heat therapeutic properties, based on Chinese medicine principles, were investigated using spectral distribution, as well as the decay probability distribution based on delayed luminescence (DL). A detection system based on ultraweak biophoton emission was developed to determine the DL decay kinetics of the cold and heat properties of Chinese herbal materials. We constructed a mathematical model to fit the experimental data and characterize the properties of Chinese medicinal herbs with different parameters. The results demonstrated that this method has good reproducibility. Moreover, there is a significant difference (*p* < 0.05) in the spectral distribution and the decay probability distribution of Chinese herbal materials with cold and heat properties. This approach takes advantage of the comprehensive nature of DL compared with more reductionist approaches and is more consistent with TCM principles, in which the core comprises holistic views.

## 1. Introduction

The cold and heat properties of Chinese medicinal herbs are classified according to the traditional Chinese medicine (TCM) principles, which comprise a core concept of TCM. The cold or heat property reflects a trend that Chinese medicinal herbs affect the transformation of heat or cold properties of the human body. Cold is a Yin disease factor that causes symptoms such as chilliness, headache, and body aches. Cold is reported to damage the Yang energy. Heat (also heat or flame) is also a Yang pathogenic factor with symptoms that include fever, inflammation, dry skin, and constipation. Chinese medicinal herbs regulate the cold or heat properties of the body to achieve a balance between them [1]. Cold herbs treat heat diseases, and heat herbs treat cold diseases. Thus, the cold or heat properties of Chinese medicinal herbs and the application of the corresponding knowledge regarding the diagnosis, differentiation, and treatment of diseases comprise important aspects in TCM. Recently, many groups have investigated herbs to identify their essence and properties [2–5]. However, the scientific evidence regarding the cold and heat properties of Chinese medicinal herbs has remained unclear. The development of a novel scientific method that provides a quantitative measure of these properties is a challenge and represents the aim of the present study.

Delayed luminescence (DL) is the long-term decay of ultraweak photon emission from biological systems following exposure to illumination, and it was discovered in green plants in 1951 [6]. DL technology has recently been used as a noninvasive tool to investigate germination, food quality, tumor cells, and environmental pollution [7–11]. DL is correlated with the functional state of the biological sample [12–15], which suggests that DL measurements may represent a potentially valuable method to analyze the cold and heat properties of Chinese medicinal herbs [16].

Despite the broad utility of DL, no study has investigated the DL signatures of Chinese herbal materials. In this study, a sensitive photon-counting system was constructed to collect DL spectra and characterize Chinese medicinal herbs [17].

In this study, we report DL data collected from the roots of Chinese medicinal herbs. The aims are to identify a correlation between the physical parameters connected to DL and the cold and heat properties of Chinese medicinal herbs as well as establish an evaluation method and indicator of the cold or heat property of Chinese medicinal herbs. This approach provides a comprehensive picture of the herbs versus the more reductionist ideas used in chromatography approaches and is more consistent with the holistic view at the heart of TCM.

## 2. Material and Methods

### 2.1. Measurement System and Measurement Procedure of DL

**Figure 1:**
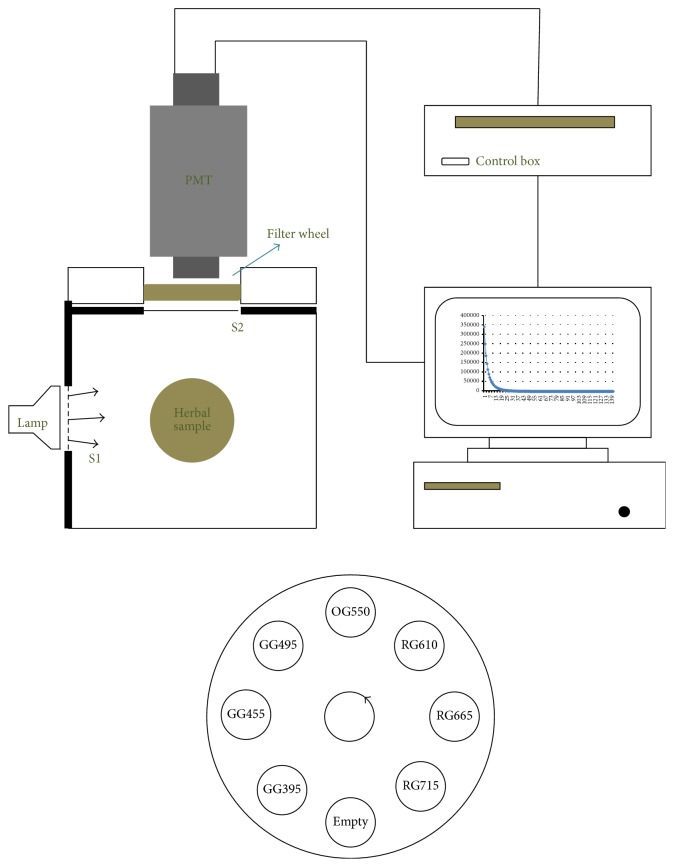
Hardware setup of spectral photon-counting system.

The device (Figure 1) includes a dark sample chamber and a vertical photomultiplier tube (PMT) with a 46 mm photocathode (Electron Tubes Enterprises Ltd., UK, type 9558QB) [17]. The PMT is cooled to −25°C to reduce the dark count rate to less than 10 counts per second.

A 55 mm Petri dish filled with the dried samples was placed in the dark chamber 12 cm from the PMT shutter. Herbs (1 g) covered the bottom of the Petri dish. To excite the samples, we used a white LED (LED Engin, USA, type LZ4-00MD00). The interval time was 100 ms. The measurement time was 20 s. A shutter system between the excitation source and the sample controlled the excitation. A photon-counting unit (HAMAMATSU, C9744) was used for all data acquisition.

During the DL measurement, a spectral analysis was performed with seven long-pass cutoff optical interference filters (Schott, Germany) [18, 19]. These filters were placed in a rotating wheel, which was located between the photomultiplier and the shutter in front of the photomultiplier. The rotating wheel has 8 openings: open (without filter), GG395, GG450, GG495, OG550, RG610, RG665, and RG715 (Figure 1). This combination of long-pass cutoff filters produces DL curves for 8 wavelength ranges: all wavelengths, <395, 395–450, 450–495, 495–550, 550–610, 610–665, 665–715, and >715 nm, respectively. The wheel rotates counterclockwise.

### 2.2. Herbal Materials

All samples (37 raw root and Rhizome herbs) were collected by Jinan Jianlian Chinese medicinal herb store in Shandong province, China. An experienced herbalist (Professor Yuanbin Zhang) at the Shandong Academy of Medical Sciences identified the samples. The samples were divided into two groups based on their cold or heat properties (Table 1).

### 2.3. Preparation of Powder

The herbal samples were pulverized to 0.125–0.177 mm with a grinder (Jinsui Company, Zhejiang province, China, type JSP-350). Different diameters of herbal particles were selected with 125, 150, and 850 *μ*m sieves (Yongkang Company, Zhejiang province, China). The sieved samples were subsequently placed in a 55 mm Petri dish and stored in a light-tight box (Chengsheng Company, Tianjin) with silica desiccant (Dingfeng Company, Zhejiang province, China, 3–5 mm Blue) for at least 16 hours prior to the DL measurements [20–23]. The water content of the samples was 6.4–7.9% according to the Chinese pharmacopoeia (2010). The room temperature was maintained at 20 ± 1°C [24–26].

### 2.4. Data Analysis

Each sample was measured at least three times, and the decay kinetic data were averaged for subsequent data analysis. Statistical analyses were conducted using SPSS V.17 software (SPSS, USA). We used an independent-sample test to compare the DL kinetic parameters of the herbal samples. *p* values less than 0.05 were considered significant. For data fitting, Statistic 10 software was used.

## 3. Results

### 3.1. DL Reproducibility Testing

**Figure 2:**
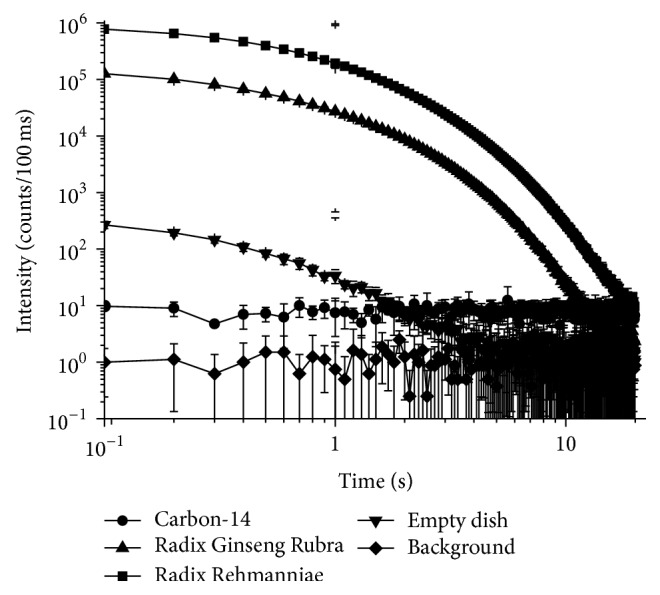
DL decay curve of* Radix Sophorae Flavescentis* (cold) and* Radix et Rhizome Ginseng Rubra* (heat) with no filter. Each sample was prepared in triplicate and analysis repeated three times. The noise of the empty dish is far lower than the signal.

We selected two representative Chinese medicinal herbs with heat and cold properties to measure the reproducibility of the DL experiments. Each sample was analyzed with the powders of five independent batches of herbs under the same experimental conditions. Empty Petri dishes served as a control (Figure 2). Figure 2 also presents the long time decay emission of two Chinese medicinal herbs (*Radix Sophorae Flavescentis* (classified as cold) and* Radix et Rhizome Ginseng Rubra* (classified as heat)) following white light illumination. The reproducibility of the technique was good. The standard deviation of the DL intensity values was <5% in the first 5 seconds and 5–15% during subsequent time points. This difference occurred because the signal was lower at the later time points; thus, the influence of the noise increased.

### 3.2. Decay Probability Distribution of DL

The kinetics of the light-induced DL temporal trends have been described by a hyperbolic function law in multiple previous studies [27–30]:

(1) $$\begin{array}{c} I\left(\;t\;\right)=\frac{I_{0}}{{\left(1+t/\tau \right)}^{\beta }}. \end{array}$$

Here, *I*_0_ is the initial intensity following illumination, *β* is the index factor associated with the rate of decay, and *τ* is the characteristic time, which is a constant specific to the sample.

Previous results implied that the DL of dried Chinese herbal materials is a complicated light emission process, which is similar to previous reports regarding sera and bacteria [31, 32]. To obtain comprehensive information regarding this complex decay process, we used a widely accepted approach in which time-resolved DL decays are described as continuous distributions of decay times or rate constants via the introduction of a probability density function *f*(*γ*) as follows [33–35]:

(2) $$\begin{array}{c} I\left(\;t\;\right)=\overset{\infty }{\underset{0}{\int }}Af\left(\;\gamma \;\right)e^{-\gamma t}d\gamma , \end{array}$$

where *A* is a normalizing constant and *γ* is a rate constant of the decay process. Based on (1) and (2), we obtain

(3) $$\begin{array}{c} \frac{I_{0}}{{\left(1+t/\tau \right)}^{\beta }}=\overset{\infty }{\underset{0}{\int }}Af\left(\;\gamma \;\right)e^{-\gamma t}d\gamma . \end{array}$$

**Figure 3:**
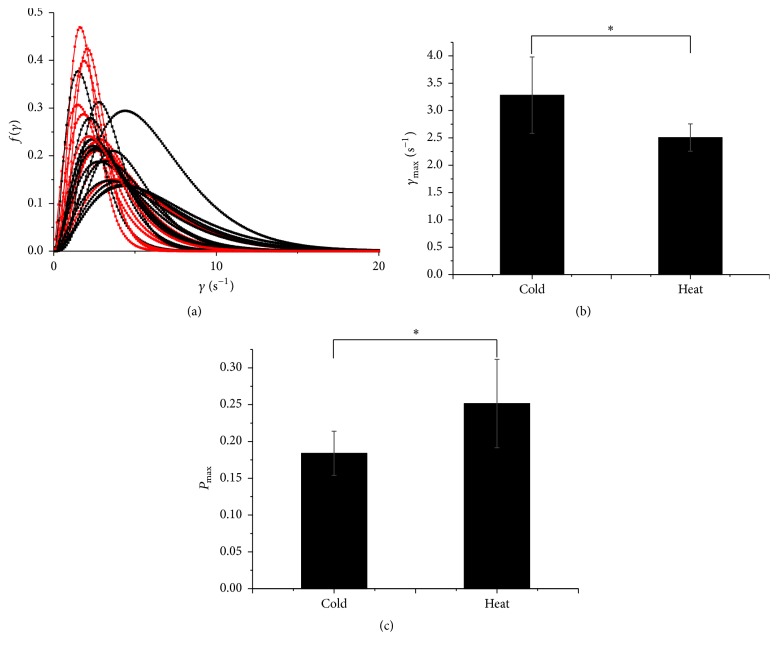
(a) The decay probability distribution in heat (red) and cold (black) herbs. (b) Comparison of the average of *γ*_max_ (23 cold herbs versus 14 heat herbs), the cold herbs average of *γ*_max_ is 3.28 ± 0.70, and the heat herbs average of *γ*_max_ is 2.54 ± 0.25; ^*∗*^ *p* < 0.05. (c) Comparison of the average of *P*_max⁡_ (23 cold versus 14 heat herbs), the average of *P*_max⁡_ is 0.18 ± 0.03 of the cold herbs, and the average of *P*_max⁡_ is 0.25 ± 0.06; ^*∗*^ *p* value < 0.05.

Therefore, the decay probability distribution *f*(*γ*) may be obtained through anti-Laplace transform processing:

(4) $$\begin{array}{c} f\left(\;\gamma \;\right)=\frac{\tau_{}^{\beta }\gamma^{\beta -1}e^{-\gamma \tau }}{\Gamma \left(\beta \right)}, \end{array}$$

where Γ(*β*) = (*β* − 1)!. Based on (4), decay probability distributions were created for 13 heat herbs and 24 cold herbs (Figure 3(a)). The cold and heat groups have substantially different decay probability distributions. The difference may also be correlated with the different ages and growth locations or postharvest processing. This leads to various complex reactions, including changes in the chemical makeup of the herbs and internal structural changes. Ultimately, external influences will be reflected in the overall efficacy of the TCM. To analyze the differences between the heat and cold Chinese medicinal herbs and to describe the DL decay kinetics, we used the peak decay rate *γ*_max_ and its corresponding peak *f*(*γ*) value. From *df*(*γ*)/*dγ* = 0, we obtain *γ*_max_ and *P*_max⁡_ as follows:

(5) γmax=β−1τ,Pmax=τγ−1β−1e1−βΓβ.

According to (1), three parameters, including *I*_0_, *β*, and *τ*, may be obtained by fitting the experimental data. According to (5), *P*_max⁡_ represents the peak value, and *γ*_max_ represents the peak decay rate. As shown in Figures 3(b) and 3(c), the average of *P*_max⁡_ is 0.18 ± 0.03 of 23 cold herbs, which is substantially less than the average of *P*_max⁡_ of 14 heat herbs (0.25 ± 0.06) and significant at *p* = 0.044. In addition, the average of *γ*_max_ is 3.28 ± 0.70 from the cold herbs which was increased compared with the heat herbs (2.54 ± 0.25) and significant at *p* = 0.03.

### 3.3. DL Emission Spectra of Chinese Medicinal Herbs

**Figure 4:**
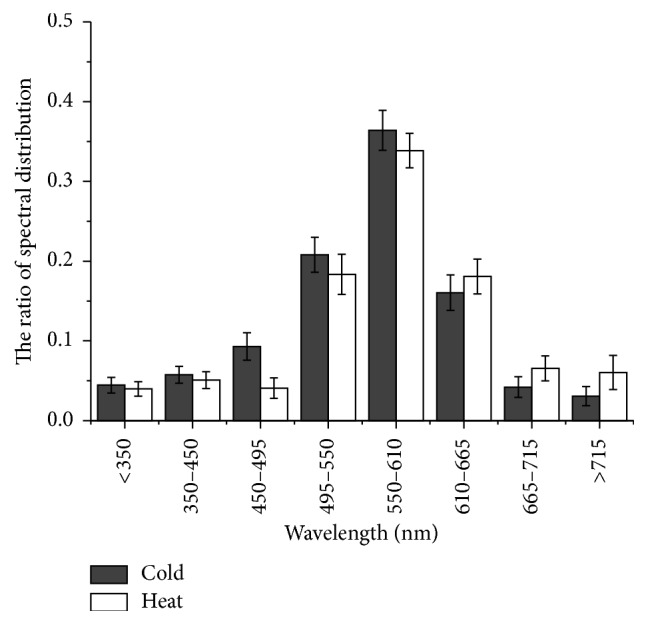
Spectral distribution of DL emission of 37 peaks found in the herbs normalized to their sum.

Another intrinsic parameter that could be used to develop a strategy of discrimination is based on the measurement of emission spectra. Our initial findings are that the DL emission spectra of different properties of Chinese herbal materials are also with obvious difference. We used 7 different long-pass filters (as described in Section 2.1) to measure DL. Different spectra were collected based on the filter set used. Along with the filter wheel rotation, the emission photons can be obtained in different spectral range depending on the filter. In order to analyze the spectral components of emission spectrum, we calculated the photon radiation of different spectral range mentioned in Section 2.1. For example, by the photons of G450 minus G395, we can get the photons spectral range 395 nm–450 nm. We normalized the spectra to compare the peak locations by dividing the photon counts at each wavelength by the total number of photons. The peak location is characteristic of the herb.  Figure 4 shows the average values and standard errors for the spectra of the heat and cold herbs. The most common characteristic spectral behavior is located from 550 to 610 nm. In comparison with the heat herbs, the cold herbs have a higher ratio range from 350 to 610 nm, and the heat herbs have a higher ratio range from 610 to 715 nm. These trends in the spectral distribution may be associated with the cold and heat properties of Chinese medicinal herbs.

## 4. Discussion

Many groups have used analytical techniques and clinical tools to investigate Chinese medicinal herbs. However, to date, modern TCM studies have not resulted in breakthroughs because of conceptual and methodological limitations. Here, we demonstrate how DL may represent an important tool to overcome some of these challenges.

The traditional analysis methods used in TCM are LC-MS and GC-MS. These approaches measure chemical compositions, including active ingredients. In general, the chemical composition governs the therapeutic properties of Chinese herbal materials. However, many earlier investigations have demonstrated that some Chinese medicinal herbs with similar chemical compositions exhibit different therapeutic properties [36, 37]. This finding may be because the entirety of the chemical repertoire in the herb is not assayed because of losses during sample preparation or low constituent abundance [38, 39]. This issue prevents a complete characterization of Chinese medicinal herbs. Moreover, traditional chemical methods emphasize a unique and specific chemical composition, which is in sharp contrast to the holistic view at the heart of TCM.

To overcome these limitations, we proposed the use of DL to characterize herbs because of its many advantages. First, it is fast, convenient, and affordable. Sample processing is limited to a simple grinding step. Samples may be immediately tested after grinding without additional chemical reagents. Moreover, very low detection limits are possible because of the sensitivity of PMTs. Importantly, this approach treats the herb as a whole, complex, and open system, which is how the body will metabolize them following consumption [40]. This approach is in sharp contrast to the reductionist approach taken in a chromatographic scheme.

## 5. Conclusion

We report a repeatable DL measurement protocol for dried Chinese medicinal herbs. We used this approach to analyze heat and cold Chinese medicinal herbs and identified a significant difference in the decay probability distribution between the two sample types. The peak decay rate (*γ*_max_) and the peak weight value (*P*_max⁡_) offered explicit discrimination between the cold and heat property herbs. The spectral behavior trends were also different and may indicate an underlying mechanism of action in TCM. Nevertheless, these findings require further validation with additional samples.

In conclusion, DL is a novel tool used to investigate materials. DL offers comprehensive information regarding both chemical constituents and energy. It is a direct, rapid, and cumulative assay that provides novel information regarding the biological nature of herbal medicines.

## Figures and Tables

**Table 1:** Nomenclature of selected cold and heat Chinese medicinal herbs.

	Pharmaceutical name	Chinese Pin Yin	*Latin botanical name *	Plant part used
Classification as cold	Radix Pulsatillae	Bai Tou Weng	*Pulsatilla chinensis* (Bge.) Regel.	Root
	Radix Dichroae	Chang Shan	*Dichroa febrifuga* Lour.	Root
	Radix Stephaniae Tetrandrae	Fang Ji	*Stephania tetrandra* S. Moore.	Root
	Radix Scutellariae	Huang Qin	*Scutellaria baicalensis* Georgi.	Root
	Radix Rhapontici	Lou Lu	*Rhaponticum uniflorum* (L.) DC.	Root
	Radix Changii	Ming Dang Shen	*Changium smyrnioides* Wolff.	Root
	Radix Curcumae	Yu Jin	*Curcuma wenyujin* Y. H. Chen et C. Ling.	Root
	Radix Arnebiae	Zi Cao	*Lithospermum erythrorhizon* Sien. et Zucc.	Root
	Radix Scrophulariae	Xuan Shen	*Scrophularia ningpoensis* Hemsl.	Root
	Radix Paeoniae Alba	Bai Shao	*Paeonia lactiflora* Pall.	Root
	Radix Trichosanthis	Tian Hua Fen	*Trichosanthes kirilowii* Maxim.	Root
	Radix Puerariae	Ge Gen	*Pueraria lobata* (Willd.) Ohwi.	Root
	Radix Panacis Quinquefolii	Xi Yang Shen	*Panax quinquefolius* L.	Root
	Radix Paeoniae Rubra	Chi Shao	*Paeonia lactiflora* Pall.	Root
	Radix Peucedani	Qian Hu	*Peucedanum praeruptorum* Dunn.	Root
	Radix Stellariae	Yin Chai Hu	*Stellaria dichotoma* L. var. *lanceolata* Bge.	Root
	Radix Sophorae Flavescentis	Ku Shen	*Sophora flavescens* Ait.	Root
	Radix Ophiopogonis	Mai Dong	*Ophiopogon japonicus* (Thunb.) Ker-Gawl.	Root
	Radix Adenophorae	Nan Sha Shen	*Adenophora tetraphylla* (Thunb.) Fisch.	Root
	Rhizoma Phragmitis	Lu Gen	*Phragmites communis* (L.) Trin.	Rhizome
	Rhizoma Anemarrhenae	Zhi Mu	*Anemarrhena asphodeloides* Bge.	Rhizome
	Rhizoma Belamcandae	She Gan	*Belamcanda chinensis* (L.) DC.	Rhizome
	Radix et Rhizome Polygoni Cuspidati	Hu Zhang	*Polygonum cuspidatum* Sieb. et. Zucc.	Rhizome and root

Classification as heat	Radix Morindae Officinalis	Ba Ji Tian	*Morinda officinalis* How.	Root
	Radix Angelicae Dahuricae	Bai Zhi	*Angelica dahurica* (Fisch. ex Hoffm.) Benth. et Hook. F.	Root
	Radix Vladimiriae	Chuan Mu Xiang	*Vladimiria souliei* (Franch.) Ling.	Root
	Radix Angelicae Sinensis	Dang Gui	*Angelica sinensis* (Oliv.) Diels.	Root
	Radix Linderae	Wu Yao	*Lindera strychnifolia* (Sieb. et Zucc.) Vill.	Root
	Radix Polygalae	Yuan Zhi	*Polygala tenuifolia* Willd.	Root
	Radix Stemonae	Bai Bu	*Stemona sessilifolia* (Miq.) Miq.	Root
	Radix Angelicae Pubescentis	Du Huo	*Angelica pubescens* Maxim. F. biserrata.	Root
	Radix Saposhnikoviae	Fang Feng	*Saposhnikovia divaricata* (Turez.) Schischk.	Root
	Radix Astragali	Hunag Qi	*Astragalus membranaceus* (Fisch.) Bge.	Root
	Rhizoma Atractylodis Macrocephalae	Bai Zhu	*Atractylodes macrocephala* Koidz.	Rhizome
	Rhizome Galanga	Gao Liang Jiang	*Alpinia officinarum* Hance.	Rhizome
	Radix et Rhizome Ginseng Rubra	Hong Shen	*Panax ginseng* C. A. Mey.	Rhizome and root
	Radix et Rhizome Asteris	Zi Wan	*Aster tataricus* L. f.	Rhizome and root
